# Resting-state brain age is associated with cognitive and sensorimotor abnormalities in schizophrenia spectrum disorders: a validation & longitudinal study

**DOI:** 10.1038/s41398-026-04426-3

**Published:** 2026-09-04

**Authors:** Sebastian Volkmer, Stefan Fritze, Dilsa Cemre Akkoc Altinok, Geva Brandt, Ersoy Kocak, Julius Wiegert, Oksana Berhe, Yuchen Lin, Heike Tost, Andreas Meyer-Lindenberg, Dusan Hirjak, Emanuel Schwarz

**Affiliations:** 1 Department of Psychiatry and Psychotherapy, Central Institute of Mental Health, Medical Faculty Mannheim, University of Heidelberg, Mannheim, Germany; 2 Hector Institute for Artificial Intelligence in Psychiatry, Central Institute of Mental Health, Medical Faculty Mannheim, Heidelberg University, Mannheim, Germany; 3 German Centre for Mental Health (DZPG), Partner site Mannheim-Heidelberg-Ulm, Mannheim, Germany

**Keywords:** Diagnostic markers, Schizophrenia

## Abstract

Brain-age models use neuroimaging features to predict chronological age. The resulting brain-age gap (BAG), defined as predicted brain age minus chronological age, quantifies deviations from age-expected brain characteristics. Structural brain-age acceleration is well established in schizophrenia spectrum disorders (SSD), but the utility of resting-state functional connectivity (rs-FC)–based brain age remains unclear. Here, we trained rs-FC brain-age models on aggregated lifespan data from healthy controls (n ≈ 2200) and evaluated them in four independent SSD case–control cohorts. Across independent cohorts and two brain atlases, SSD showed higher FC-based BAG than healthy controls (β ≈ 0.4–0.6), indicating modest functional brain-age elevation at the group level. However, within SSD, more negative (younger appearing) BAG was associated with poorer cognitive performance, longer duration of illness, and higher neurological soft signs (NSS). Over 12–24 weeks, increases in BAG were associated with reductions in NSS motor coordination and hard signs. Together, these findings suggest that rs-FC brain age captures both a small case–control shift and a clinically relevant dimension within SSD that is not well described by uniform “acceleration”. FC-based BAG may therefore reflect heterogeneity in network-level development and reorganization, with younger-appearing functional profiles potentially indexing greater neurodevelopmental burden.

## Introduction

Estimating “brain age” from neuroimaging has emerged as a compact way to summarize age-related brain characteristics and quantify deviations from age-expected patterns that may be associated with disease [1]. Related normative-modeling work explicitly frames these trajectories as growth-chart–like reference distributions, enabling subject-level deviation scores that can be linked to psychopathological symptoms, cognition, and prognosis in psychiatry [1]. In parallel, functional-connectivity (FC)–based brain-age models trained on large, multi-site resting-state fMRI (rs-fMRI) datasets show that multiscale connectivity is informative for aging and that cross-site harmonization materially improves prediction [2]. However, effect sizes vary by sex and decrease with age [3].

Schizophrenia spectrum disorders (SSD) are a valuable benchmark for brain-age modeling. Mega-analyses across 26 international cohorts indicated patients’ brains appear, on average, several years “older” than their chronological age when using structural MRI features, although relationships with psychopathological symptoms or medication were inconsistent at scale [4]. The brain-age-gap (BAG) refers to the difference between an individual’s predicted brain age and their chronological age, providing an index of accelerated or delayed brain aging. Region-wise and multimodal studies converge on prominent structural contributions, particularly within frontal, temporal, and insular territories, to elevated BAG in SSD [5]. By contrast, evidence that FC-based brain-age estimates reflect accelerated functional brain aging in SSD is mixed: a recent multicenter study constructing regional brain-age models across modalities reported robust structural (gray/white matter) acceleration but no FC-based acceleration [5]. Similarly, a multimodal neuroimaging study in early psychosis similarly found stronger relationships between clinical status and structural BAG than with functional brain age [6]. Other approaches, however, have identified FC signatures in young individuals with subclinical psychotic symptoms [7], and recent work has suggested that brain-age models with lower overall accuracy may nevertheless exhibit greater sensitivity for disease detection [8]. Additionally, longitudinal research on BAG in SSD remains limited. For example, Fan and colleagues investigated the relationship between structural BAG and 12-week treatment outcome and identified BAG as a predictor of the functional outcome [9] However, their study included a relatively modest sample (n = 49) and did not investigate longitudinal changes in BAG. Consequently, it remains unknown whether brain-age estimates themselves change over time and whether such changes parallel clinical trajectories. Longitudinal rs-fMRI–derived BAG measures would provide the opportunity to determine whether within-person changes in functional BAG track within-person changes in psychopathological symptoms. Because rs-fMRI exhibits both stable and dynamic components [10], repeated rs-fMRI assessments could further clarify whether ongoing network reorganization is reflected in longitudinal BAG changes or whether functional BAG primarily represents a stable neurodevelopmental trait marker.

Two methodological issues are pivotal for FC-based brain-age examination. First, the choice of atlas/parcellation changes both the extracted connectome and downstream prediction. Recent benchmarks and cross-atlas evaluations showed that parcellation granularity and atlas type can affect reproducibility and accuracy [11, 12]. In the present study, we selected two commonly used but conceptually distinct parcellations: the Harvard-Oxford atlas [13–16], an anatomically defined atlas with clinically interpretable cortical and subcortical regions, and the Multi-Subject Dictionary Learning (MSDL) atlas [17], a functionally derived atlas capturing large-scale resting-state networks. Together, these atlases capture complementary representations of brain organization and allowed us to determine whether the resulting brain-age estimates were consistent across anatomical and functional parcellation strategies. In addition, both atlases yield moderate-dimensional connectivity feature spaces relative to higher-resolution alternatives such as Schaefer-[18] or Brainnetome-based [19] parcellations, thereby reducing the risk that atlas choice primarily reflects dimensionality-driven differences in model complexity when comparing multiple machine-learning algorithms across preprocessing strategies and independent validation cohorts. Second, pipeline decisions (region definition, connectivity estimation, learning algorithm) can each modulate predictive performance, motivating transparent, side-by-side model comparisons rather than reliance on a single method [20]. Despite this, systematic evidence remains limited on how widely used FC atlases perform when combined with comparatively traditional and interpretable machine-learning models, even though such models are attractive for clinical translation because of their stability, transparency, and modest computational demands. These design choices are especially critical when targeting modest effect sizes and the need to generalize across scanners, sites, and clinically heterogeneous populations.

Here, we address these gaps by training and evaluating rs-fMRI brain-age models in a large normative cohort (n = 2222), systematically comparing two commonly used FC parcellations and a diverse panel of conventional machine-learning models (regularized linear models, kernel methods, and gradient-boosting ensembles): First, we identify which atlas-by-model combinations most accurately predict age and reproduce across preprocessing strategies. Second, we evaluate generalization to four independent cohorts including healthy controls (HCs) and individuals with SSDs, quantifying BAG group differences and examining associations with psychopathological symptoms, cognitive functioning, neurological soft signs (NSS), daily antipsychotic medication dose, and illness duration. Finally, in our in-house longitudinal SSD cohort, we investigate whether BAG predicts changes in psychopathological symptoms, cognitive functioning, and NSS over a 12-week and 24-week follow-up period. This framework provides a baseline for FC-based brain-age modeling and clarifies when and how lower-complexity models (e.g., linear/regularized) can yield sensitive, generalizable markers of disease-related brain aging. We expect, compared with HCs, individuals with SSDs will show higher brain-age gap (older-appearing brains). Nonlinear machine-learning models will outperform linear models in rs-fMRI age prediction. FC–based BAG will be larger than previously reported structural MRI brain-age effects.

## Methods

### Data

We analyzed four deeply phenotyped inhouse and two publicly available clinical datasets comprising individuals with SSD and HCs. In parallel, we assembled an independent normative reference cohort by aggregating HCs from six openly available studies. Cohort characteristics, recruitment criteria, imaging protocols, and quality-control procedures are detailed below and in the Supplementary Information. For the clinical cohorts, held-out HC participants were used to estimate site effects and avoid confounding the BAG calculation. For the in-house datasets BMBF and URBN, these participants were presplit; for the other two cohorts, a hold-out set was selected as described in Section 2.4.

#### In-house clinical datasets (whiteCAT, URBN, NSS, BMBF)

We analyzed four in-house datasets—whiteCAT, URBN, NSS and BMBF—comprising individuals with SSD and HCs. All participants provided written informed consent after a full explanation of study procedures. SSD participants were evaluated during in- or outpatient treatment shortly after partial remission of acute psychopathological symptoms. Core assessments (neuroimaging, psychopathology and neuropsychology) were completed within seven days, and patients were required to be on a stable daily dose of antipsychotic or antidepressant medication for at least seven days before inclusion. Information about the MRI data acquisition can be found in the Supplement Section 1.1.1.

##### Data integration for case–control analyses

Because NSS contained only SSD patients and whiteCAT a small subset of HCs and SSD patients, whereas URBN and BMBF comprised only HCs, we paired cohorts acquired on the same scanner with identical imaging protocols and merged them to create two independent case–control datasets: whiteCAT+URBN and NSS + BMBF.

##### Cohort descriptions

**whiteCAT (SSD + HC)**: 90 SSD patients and 18 HCs were recruited from the in- and outpatient services of the Central Institute of Mental Health (CIMH), Mannheim, Germany, as part of a larger cohort (see Hirjak et al. [21]). Diagnoses were established clinically by GAB, SF and DH. Eligibility included adults aged 18–64 years with ICD-11 schizophrenia or other primary psychotic disorders (codes 6A20–6A25), with or without psychotic symptoms. Exclusion criteria were inability to speak German, known cognitive disability (IQ < 70) or dementia, substance use disorder in remission for <12 months, and neurological or medical conditions that could affect the study constructs. The study was approved by Ethics Committee II, Medical Faculty Mannheim at Heidelberg University, Germany.

**URBN (HC):** HCs aged 19–53 years were enrolled at CIMH, Mannheim. Participants were prospectively assigned to training (n = 75) and validation (n = 25) sets. Individuals with any prior mental or neurological diagnosis were excluded at screening. All participants underwent clinical assessment (SKID-II interview, DSM-5) to confirm the absence of psychiatric conditions (current; no treatment, no medication). The exclusion criteria (at screening) - general medical issues, neurological illness, MRI contraindications, a history of head trauma, and substance dependency; for both sub-samples.

**NSS (SSD)**: 129 SSD participants met DSM-IV criteria [22]. Diagnoses were made by staff psychiatrists and confirmed with the German versions of the Structured Clinical Interview for DSM-IV Axis I and II Disorders (SCID) and review of case notes (reviewed by SF and DH).

**BMBF (HC)**: 246 HCs aged 18–58 years were enrolled at CIMH, Mannheim [23], with a priori allocation to training (n = 221) and validation (n = 25) sets. Individuals with any prior SSD diagnosis were excluded at screening.

##### Clinical and cognitive measures

Psychopathology in SSD was quantified with the Positive and Negative Syndrome Scale (PANSS [24]). For this analysis four scores of the PANSS were investigated, the total score, positive symptom score, negative symptom score, and general symptoms score. Cognitive functioning was measured with the Brief Cognitive Assessment Tool (B-CATS [25]). This cognitive test included a trail-making test (TMT), digit-symbol-substitution-test (DSST), and categorical fluency (CF). Demographic and clinical characteristics are reported in Supplement Tables 1 and 2. Medication was assessed with olanzapine equivalence calculation based on the dosages by Leucht et al. [26]. NSS were evaluated with the Heidelberg NSS scale [27]. For this analysis, we evaluated the NSS total score and its five subdomains Motor coordination (MoCo) that was evaluated with five tasks—Ozeretski’s test, diadochokinesia, pronation–supination, finger-to-thumb opposition, and speech articulation. Integrative functions (IF) that was assessed with three tasks—standing and gait, tandem walking, and two-point discrimination. Complex motor tasks (CoMT) that was measured with two tasks—finger-to-nose and fist–edge–palm. Right/left and spatial orientation (RLSpO) that was examined with four tasks—right/left orientation, graphesthesia, face–hand test, and stereognosis. Hard signs (HS) that were assessed with two tasks—arm-holding test and mirror movements.

##### Longitudinal assessment

SSD patients in the whiteCAT and NSS cohorts were assessed twice, at baseline and after 12 (whiteCAT) and 24 (NSS) weeks of treatment as usual (TAU) according to their psychiatrists’ choice, to obtain a precise, comprehensive, and longitudinal collection of clinical (e.g., psychopathological symptoms, cognitive functioning and NSS) and neuroimaging data, with the goal of predicting clinical response over the 12/24-week treatment period.

#### Publicly available clinical datasets (LA5c-UCLA, COBRE)

##### Cohort descriptions

**LA5c-UCLA (OpenNeuro, accession ds000030):** The UCLA Consortium for Neuropsychiatric Phenomics LA5c study provides multimodal MRI data from 272 participants (HCs, n = 130; clinical groups including SSD, n = 50; ages 21–50 years). HCs were recruited via community advertisements and were required to have ≥8 years of education, fluency in English or Spanish, and no major psychiatric disorders (including schizophrenia, bipolar disorder, ADHD, substance abuse, or major depression). SSD participants meeting the same age and health criteria were recruited via targeted clinical outreach and online platforms. All participants provided written informed consent under protocols approved by UCLA and the Los Angeles County Department of Mental Health; individuals undergoing fMRI were additionally screened for MRI safety [28]. Information on MRI data acquisition can be found in the Supplement Section 1.1.2.

**COBRE (via SchizConnect):** The Center of Biomedical Research Excellence (COBRE) [29] dataset includes structural MRI for 186 participants (HCs, n = 91; SSD, n = 85; ages 18–66 years). Diagnoses were established with the Structured Clinical Interview for DSM Disorders; the SSD group comprised schizophrenia (n = 74) and schizoaffective disorder (n = 11) [30]. Exclusion criteria included neurological disorders, head trauma with loss of consciousness >5 min, and substance abuse within the preceding 12 months. Diagnostic categories not meeting the study’s inclusion criteria (e.g., bipolar disorder) were excluded from the present analyses. All participants gave written informed consent under local institutional review board approvals. Information on MRI data acquisition can be found in the Supplement Section 1.1.2.

#### Healthy Reference Cohort

We constructed a healthy reference cohort of HCs from six distinct publicly available datasets. Cohort characteristics, recruitment criteria, imaging protocols, and quality-control procedures are detailed below and in the Supplementary Information.

**CamCAN (accessed via the CamCAN CBU Data Portal):** The Cambridge Centre for Ageing and Neuroscience (CamCAN) repository, a population-based, cross-sectional adult lifespan sample (653 HCs; 18–87 years). The dataset comprises multi-modal neuroimaging, including sMRI and rs-fMRI [31].

**AOMIC-PIOP (OpenNeuro, 1: accession ds002785; 2: accession ds002790):** Two datasets were utilized from the Amsterdam Open MRI Collection (AOMIC), that contained multimodal MRI data, i.e., sMRI and rs-fMRI data, from a Population Imaging of Psychology (PIOP), with PIOP1 216 HCs from the age of 18-26 and PIOP2 226 HCs from the age of 18-25 [32].

**DLBS (OpenNeuro, accession ds004856):** The Dallas Lifespan Brain Study (DLBS) is a longitudinal, multimodal investigation of adult brain aging initiated in 2008 and consists of three waves. It includes sMRI and rs-fMRI of 464 individuals from the age of 20-90 [33]. Since we did not want to bias the cohort for specific subjects that underwent all three waves of the longitudinal study, we included only unique subject labels and data from their first visit. For example, if a subject appeared in all three waves, we only utilized data from the first wave.

**OASIS-3 (accessed via NeuroImaging Tools & Resources Collaboratory (NITRC):** The third series of the Open Access Series of Imaging Studies (OASIS-3) is a retrospective aggregate dataset comprising 1378 participants containing sMRI and rs-fMRI data. The cohort includes 755 cognitively normal adults and 622 individuals spanning various stages of cognitive impairment, with ages ranging from 42-95 years [34]. Since this data was utilized for the HC reference, only 755 cognitively normal adults were included for our brain age modeling. The subjects underwent multiple T1w scans, however, to stay consistent with the other dataset, we only included one of the T1w scans, i.e., the scan with the lowest run number that was not corrupted.

**SALD (accessed via the 1000 functional connectome project (1000fcon)):** The Southwest University Adult Lifespan Dataset (SALD) consists of sMRI and rs-fMRI data from 494 HCs aged 19-80 years [35].

### Brain connectivity calculation

All MRI data were preprocessed using fMRIPrep version 23.2.3 [36], more details on the preprocessing steps with fMRIPrep can be found in the Supplement. The connectivity matrices were calculated with nilearn version 0.10.2 [37] on the python version 3.9.18. The connectivity matrices was calculated using two atlases: the Harvard-Oxford atlas consisting of 48 cortical unilateral and 11 unilateral (7 were considered in this study) subcortical structural areas [13–16], resulting in total of 69 bilateral brain regions (brain stem is not divided into left & right), and the Multi-Subject Dictionary Learning (MSDL) atlas from nilearn, derived from rs-fMRI [17]. The MSDL atlas is a probabilistic functional atlas derived from rs-fMRI using multi-subject sparse dictionary learning. It comprises 39 spatial maps corresponding to coherent patterns of spontaneous functional activity, including large-scale functional networks such as default mode, attentional, visual, auditory, sensorimotor, and subcortical components. Thus, whereas the Harvard-Oxford atlas provides an anatomically defined parcellation, the MSDL atlas provides a complementary functionally defined representation of intrinsic brain organization [17]. The MSDL atlas was previously used in studies predicting schizophrenia [38].

For each of these atlases we derived region-of-interest (ROI) time series from rs-fMRI. Preprocessed images were taken from the project’s fMRIPrep derivatives (version 23.2.3; MNI152NLin2009cAsym space; 2 mm isotropic resolution). Time- series extraction was employed with the NiftiMapsMasker. Prior to extraction, signals were (i) detrended and (ii) standardized within runs (z-scored; standardize = ‘zscore_sample’).

Confound regression was performed using nilearn’s fMRIPrep interface (*load_confounds_strategy*) with the preset denoising strategy *denoise_strategy* = *‘simple’*. This preset implements a commonly used resting-state fMRI denoising strategy based on Fox et al. [39] and includes high-pass filtering, a full motion model, and basic white-matter and cerebrospinal-fluid regressors. In the present analysis, we additionally included a basic global signal regressor (*global_signal* = *‘basic’*). The full motion model includes the six rigid-body translation and rotation parameters, their temporal derivatives, quadratic terms, and squared derivatives, resulting in 24 motion-related regressors. All selected confounds were supplied to the masker during time-series extraction.

Head motion was therefore accounted for at two stages: during time-series extraction through regression of fMRIPrep-derived motion parameters, and in the statistical models through inclusion of mean Framewise Displacement (FD) as a subject-level motion covariate. Mean FD was selected as the subject-level motion summary because FD provides a widely used estimate of volume-to-volume head motion in resting-state fMRI analyses, consistent with other rs-fMRI studies [40, 41]. Quality control was done manually by investigating the output of fmriprep, including the alignment of grey and white matter boundaries of the T1w image and carpet plots of the functional MRI. Additionally, every subject was dropped that exceeded mean FD of 0.5 mm to remove subjects with excessive head motion during the measurement. This threshold was used in previous SSD rs-fMRI studies [42]. This resulted in the following subjects dropped due to excessive motion: AOMIC1: n = 7; AOMIC2: n = 12; CamCan n = 62; OASIS n = 108; SALD n = 10; DLBS n = 6; whiteCAT n = 4; URBN n = 1; NSS n = 16; BMBF n = 6; COBRE n = 44; UCLA n = 8.

For the Harvard-Oxford atlas we merged the cortical and subcortical atlas, while also dropping regions/labels of non-interest, i.e., left & right cerebral white matter, left & right cerebral cortex, left & right lateral ventricle, and brain stem (unilateral n = 4; bilateral n = 7). The connectivity matrix was calculated with the ConnectivityMeasure object from nilearn with Pearson correlations and standardized with ‘zscore_sample’. From each atlas we only take the unique connectivity values for further analyses, i.e., the lower triangular matrix. This results in the following number of features MSDL: 741 features; Harvard-Oxford: 5995 features (110 × 109 / 2 unique pairwise connections).

### Clinical cohort matching

We performed prospective matching to align the age and sex distributions of HCs and participants with SSD participants. Although the whiteCAT/URBN and NSS/BMBF cohorts already included external validation sets, we additionally matched participants within each cohort. For NSS/BMBF, we computed propensity scores via logistic regression (predictors: age and sex) and implemented k-nearest neighbor (kNN) matching using the PsymPy package [43]. We applied the same procedure to the UCLA and COBRE datasets, which lacked dedicated validation sets for subsequent site harmonization.

In whiteCAT/URBN, the number of HCs was smaller than the number of SSD participants, necessitating a reduction of the SSD sample to achieve comparable distributions. Specifically, we estimated propensity scores with a logistic regression model including age and sex, then performed kNN matching with a caliper of 0.3 standard deviations on the logit of the propensity score; SSD participants without an eligible HCs match within this caliper were excluded, for all calculations that looked at group effects between HCs and SSD participants.

### Site harmonization & confounding

We harmonized FC across sites with the neuroHarmonize implementation [44] of ComBat [45], specifying age, sex, and mean FD as preserved covariates. To avoid bias in group comparisons and clinical and cognitive associations, the ComBat model was trained exclusively on an independent healthy-reference pool comprising the whiteCAT/URBN and NSS/BMBF validation sets as well as the UCLA and COBRE HCs excluded during propensity-score matching. All downstream statistical analyses were therefore conducted on data unseen by the harmonization model. After ComBat, we further removed nuisance variance by fitting a linear model and retaining the residuals after correcting for sex and mean FD.

### Estimating biological brain age

For each brain atlas (MSDL, Harvard-Oxford) and each connectivity calculation (pearson correlation) different Machine Learning (ML) models were fitted to estimate the biological brain age. Seven model families (Support Vector Regression (SVR), Elastic Net, Ridge Regression, Lasso Regression, Random Forest, with scikit-learn version 1.3.2 [46] LightGBM version 4.3.0 [47], XGBoost version 2.0.3 [48]) were tuned with optuna hyperparameter (Bayesian optimization) [49] search inside a nested cross-validation (outer with five folds and five repeats stratified by age decade; inner 3 folds). Performance on outer test folds was summarized with Mean Absolute Error (MAE), Root Mean Squared Error (RMSE), R², and Pearson’s r to the biological age; 95% CIs were computed using t-based intervals (for MAE/RMSE/R²) and Fisher z-transforms (for r). The best-performing family by mean MAE across outer folds was identified and refit on all HC data with the best hyperparameters from the corresponding outer fold to yield the final biological age predictor. Lastly, the raw predictions of brain age models tend to overestimate in the young und underestimate in the old population (regression towards) mean, that’s why applied an additional adjustment for the intercept and slope (substract by the intercept and divide by the slope) of the original ML model [50]. More information can be found in the Supplement 1.2.

### Statistical analyses

**Fig. 1:**
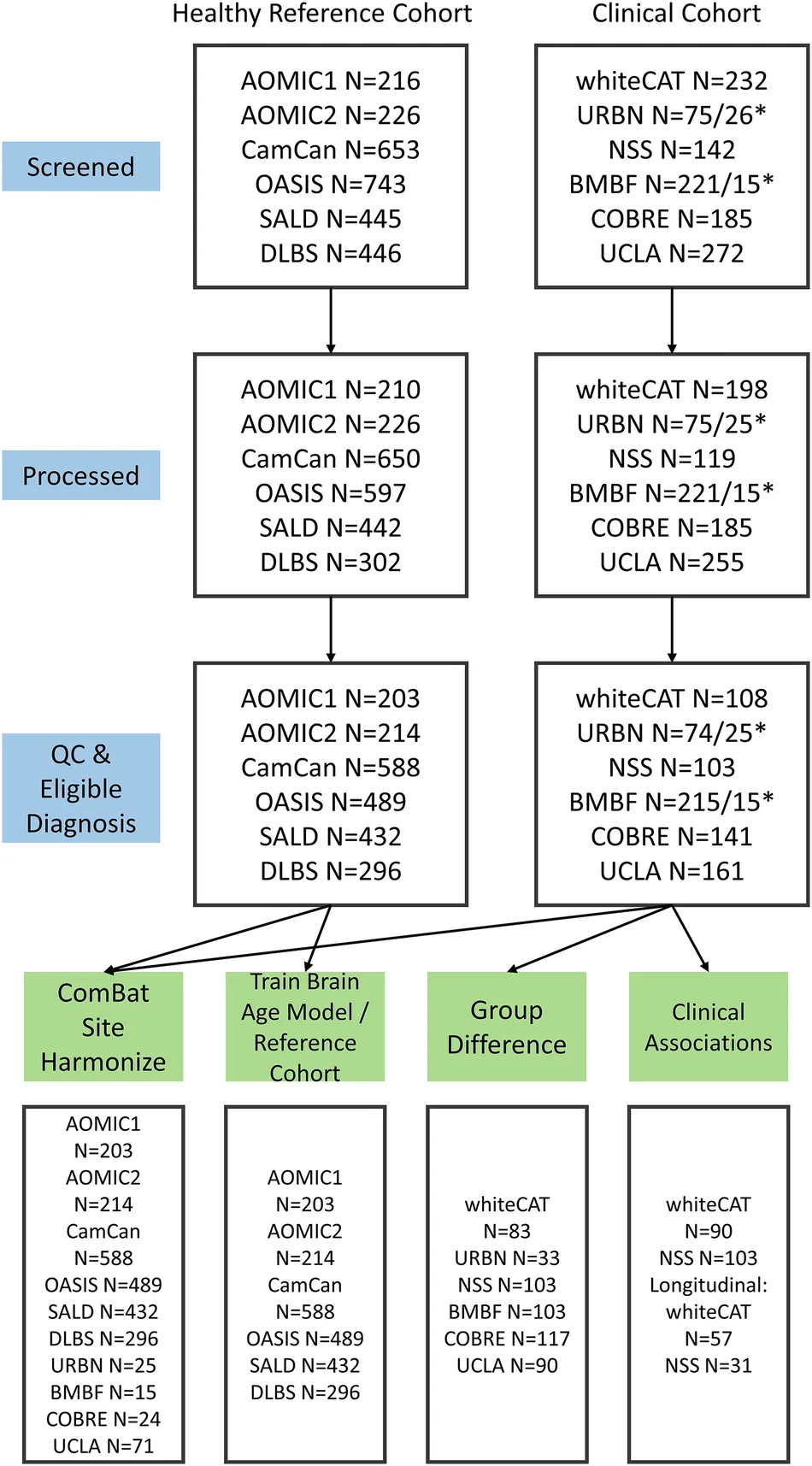
Flowchart of participant inclusion across the analysis steps. “Screened” refers to all participants who underwent an MRI session. “Processed” refers to participants with complete MRI data who successfully completed preprocessing. “QC & Eligible Diagnosis” refers to participants retained after quality-control procedures and exclusion of diagnostic labels that were not relevant to the present analyses. For the UCLA dataset specifically, participants were excluded if “ghosting artefacts” were reported in the metadata. During the “QC & Eligible Diagnosis” step, the following numbers of participants were excluded after QC: AOMIC1, n = 7; AOMIC2, n = 12; CamCAN, n = 62; OASIS, n = 108; SALD, n = 10; DLBS, n = 6; whiteCAT, n = 4; URBN, n = 1; NSS, n = 16; BMBF, n = 6; COBRE, n = 44; UCLA, n = 8. The asterisk (*) indicates the split between training and validation datasets.

BAG was first residualized with respect to key demographic and motion covariates. Specifically, on sex, mean FD, age (linear term), and age² using a linear model. The residuals from these models were then converted to standardized z-scores within the pooled sample and used as dependent variables in all analyses. All analyses were corrected for multiple hypothesis testing using the FDR correction of Benjamini-Hochberg (alpha = .05) [51]. Figure 1 shows the flow-chart of different cohorts that were used for each analysis.

#### HC & SSD group difference

We used an ordinary least-squares linear regression model with site modeled using fixed effects. The design matrix thus included an intercept, diagnosis (SSD = 1, HC = 0), sex, centered age, centered age squared, centered mean FD, and site variable. To obtain inference that is robust to heteroscedasticity, we estimated standard errors using the HC3 estimator [52]. For the diagnosis term, we report the regression coefficient (β), its 95% confidence interval (CI), and the two-sided p value. To complement this analysis Welch’s t-test was performed for each clinical site separately with z-scores only computed in that site [53]. For this analysis group means, standard deviations are reported. Effect sizes for each site were summarized as Cohen’s d [54] and Hedges’ g [55]. This analysis was conducted in Python with statsmodels version 0.14.1 [56].

#### Clinical association

Each clinical variable was first harmonized and transformed using a robust rank-based inverse normal transformation [57]. Specifically, within the pooled sample we converted the observed values to average ranks, mapped ranks to empirical cumulative probabilities, and then transformed these probabilities to standard normal deviates (z-scores) using the inverse error function. The resulting clinical z-scores were then re-standardized to have unit variance (SD = 1), such that all regression coefficients can be interpreted as the change in the brain outcome per 1 SD increase in the transformed clinical measure. For each association, an ordinary least-squares regression model with site included as a fixed effect and HC3 robust standard errors was fitted with BAG as the outcome variable. For each association, we report the standardized regression coefficient (β per 1 SD increase in the clinical variable), its standard error, the 95% confidence interval, the two-sided p value, and corrected q value.

#### Longitudinal outcome

Longitudinal BAG and clinical outcome was calculated as ${Variable}\Delta ={{{{\rm{x}}}}}_{{{{\rm{fu}}}}}-{x}_{{baseline}}$. Here clinical variables were also rank-based inversed transformed and the associations were again, fit with a GLM with a HC3 estimator.

All above mentioned methods were performed in accordance with the relevant guidelines and regulations.

## Results

### Demographic and clinical characteristics

Demographic characteristics of all study cohorts are shown in Supplement Table 1 & 2. Supplement Table 2 shows significant group differences calculated via a two-sided t-test in age (t = 3.07, p = .002) in the NSS/BMBF cohort and in mean FD (t = -2.93,p = .004) in the UCLA cohort and NSS/BMBF cohort (t = -4.7,>p = .001). No significant differences in sex were observed using chi-squared tests. Specific clinical characteristics of the two in-house cohorts are shown in Supplement Table 3. Supplement Table 4 shows the clinical characteristics at baseline and follow-up of patients that had a second visit.

### Brain age estimation model selection

**Fig. 2:**
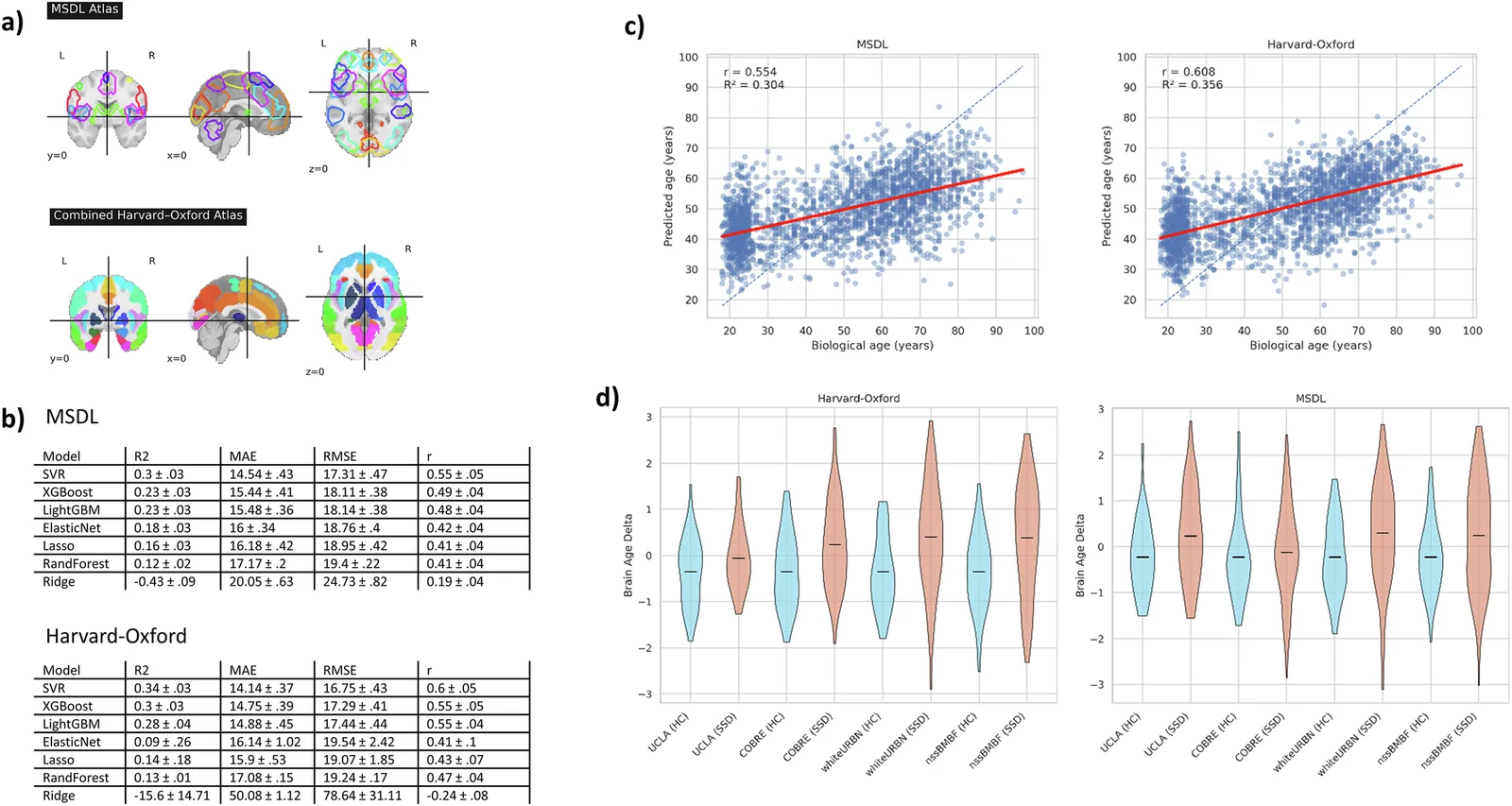
Brain age prediction and diagnostic group difference. **a** Shows the two different parcellations, the functional MSDL atlas and the structural cortical and subcortical Harvard-Oxford atlas. **b** Shows the performance measures of each ML model during the nested CV for hyperparameter optimization. **c** Shows the regression plot of the predicted age of the final model (SVR with an RBF kernel in both atlases) on the hold-out-set when trained on the healthy reference cohort against the true biological age. **d** Shows the group differences of BAG for each site in the four clinical cohorts.

As shown in Fig. 2, for both the MSDL and Harvard-Oxford atlas, the best performing model was the Support Vector Regression (SVR) with an RBF kernel, followed by XGBoost. Generally, SVR (MSDL MAE: 14.54; Harvard-Oxford MAE: 14.14) and tree boosting algorithms performed better compared to the linear models and random forest. Additionally, SVR, LightGBM, and XGBoost, performed better on the Harvard-Oxford atlas while the other models performed better on the MSDL atlas. In the clinical cohorts SVR performed similar with a mean MAE of 13.45 in the MSDL atlas and a mean MAE of 14.35 in the HC group and 14.88 MAE (MSDL) 16.04 MAE (Harvard-Oxford) in the SSD group.

### Group difference analysis in the clinical cohort

Figure 2d depicts the group differences in BAG for each of the selected atlases. BAG shows the same pattern regardless of cohort or atlas. Specifically, BAG was higher in individuals with SSD compared to HC when using the Harvard–Oxford atlas in (β = 0.62, CI 0.46–0.79, p = 1.9e-13), and when using the MSDL atlas (β = 0.39, CI 0.21–0.56, p = 1.1e-5).

Supplementary Tables 5 (MSDL) and 6 (Harvard–Oxford) show consistently higher BAG in patients than controls across sites, largely independent of atlas choice; the only exception was the COBRE cohort using the MSDL atlas, where no significant difference was observed.

### Clinical associations

**Fig. 3:**
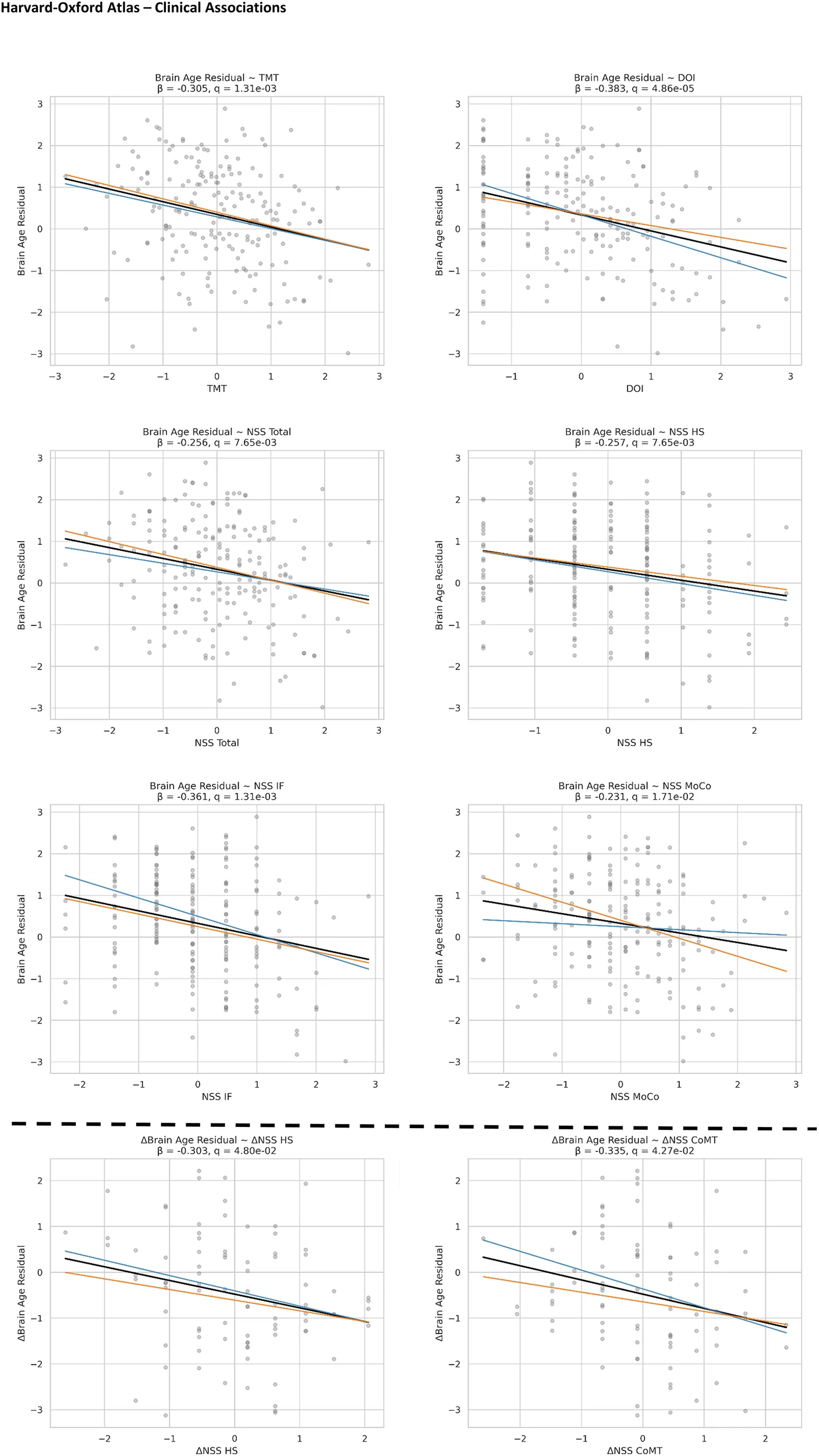
Shows the regression plots of the clinical associations with the Harvard-Oxford atlas. The blue line indicates the whiteCAT cohort, the orange line indicates the NSS cohort, and the black line represents both cohorts. Significant longitudinal outcome changes are shown below the dashed line.

**Fig. 4:**
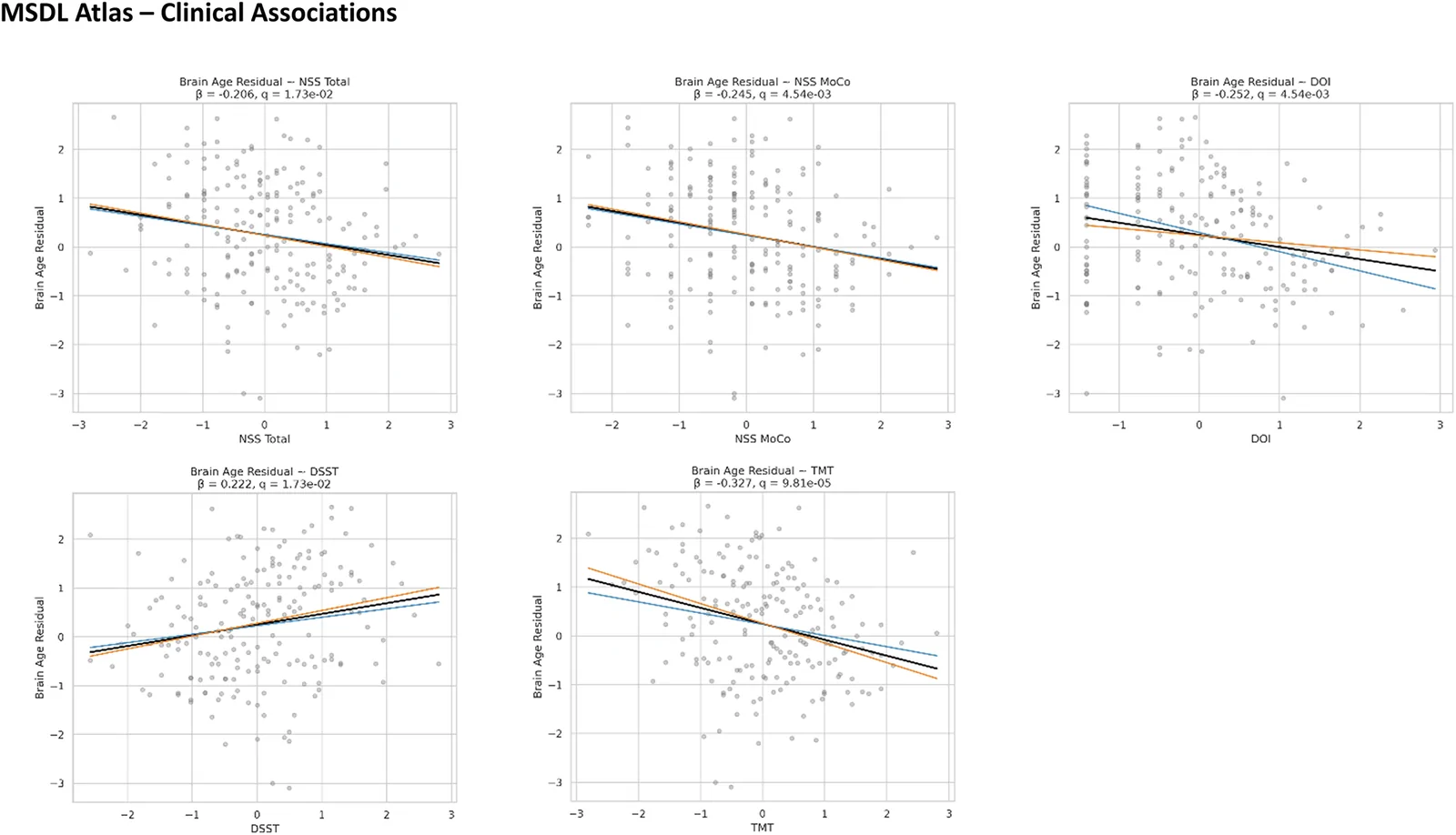
Shows the regression plots of the clinical associations with the MSDL atlas. The blue line indicates the whiteCAT cohort, the orange line indicates the NSS cohort, and the black line represents both cohorts.

Figure 3 and Fig. 4 show the associations between BAG and clinical variables for each atlas. Regardless of atlas, a younger-appearing brain was associated with higher NSS scores, longer DOI, higher TMT, and lower DSST. For the Harvard–Oxford atlas, this means that an older-appearing brain was associated with lower TMT time, shorter DOI, and lower NSS scores (HS, IF, RLSpO, Total). For the MSDL atlas, the pattern was similar: older-appearing brains were associated with better TMT and DSST performance, shorter duration of illness, and lower NSS scores (RLSpO, Total).

### BAG and clinical variables change over time

In the whiteCAT and NSS SSD cohort, an increase in BAG was associated with a reduction in the NSS subscale scores for MoCo and HS, but this associations was only shown with the Harvard-Oxford atlas (HS β = -0.3, CI − 0.52–-0.08, q = 0.007; MoCo β = -0.33, CI − 0.56–-0.11, q = 0.003). Figure 3b visualizes these results.

### Post-Hoc analysis: group difference between younger and older appearing patients

Since our findings showed that a smaller and negative BAG was associated with a worse clinical outcome, and a positive change of BAG was associated with an NSS symptom improvement at FU, a post-hoc analysis was conducted. Here, we performed a Welch t-test between the SSD patients that appeared at least 0.5 SD younger against the SSD group that appeared 0.5 SD older. The results can be found in Supplement Table 7 for the MSDL atlas and in Supplement Table 8 for the Harvard-Oxford atlas. In this analysis, in both atlases we found that more SSD patients independent of atlas choice appear at least 0.5 SD older. In line with our previous analysis, we found a mostly negative trend, and that patients in the group of 0.5 SD younger brain age had worse clinical ratings and cognitive scores. In both atlases a significantly higher DOI and TMT score, in the MSDL atlas a significantly higher NSS MoCo, and total score, and in the Harvard-Oxford atlas a significantly higher NSS HS, and IF score were associated with younger brains.

## Discussion

This study integrates publicly available datasets with deeply phenotyped in-house longitudinal cohorts to investigate FC-based brain-age models in SSD. It offers three main contributions: First, we showed that purely rs-FC–based brain-age models trained on a large, harmonized lifespan reference detect modest but robust functional brain-age elevation in SSD across four independent cohorts, echoing structural MRI work from ENIGMA and others that reports ~3–5 years of structural over-aging in SSD [4]. This indicates that large-scale connectivity patterns alone carry an age signal that is sensitive to disease-related deviation, even at typical rs-fMRI scan lengths and in clinically heterogeneous samples.

Second, we found that SVR with an RBF kernel outperformed other ML models for biological age prediction: across both MSDL and Harvard–Oxford parcellations, SVR consistently outperformed linear models and tree-based ensembles, while remaining computationally lightweight and interpretable. This aligns with recent benchmarking neuroimaging studies where shallow kernel methods remain competitive with, or superior to, more complex algorithms for brain-age prediction when data are multi-site and harmonized [58]. Together with our atlas comparison, this provides a pragmatic template for future translational work: standard FC atlases plus SVR can deliver robust, generalizable brain-age estimates without bespoke deep-learning infrastructure.

Third, we showed that within SSD, delayed-maturation appearing functional brains were associated with higher clinical scores. A more negative BAG was linked to reduced global cognition, longer DOI, and higher NSS across several subdomains. Importantly, this within-group pattern is not inconsistent with the modestly elevated BAG observed at the case–control level. BAG is a deviation score derived from a single normative age axis, and heterogeneity in SSD may yield deviations in both directions: some patients may show “older-like” FC patterns consistent with illness-related network inefficiency, whereas others may show “younger-like” patterns reflecting delayed, atypical, or less differentiated functional organization. Under this heterogeneity model, a lower functional BAG likely does not indicate preserved youthfulness; rather, it may index departures from normative maturation that the age-prediction model maps to younger ages. This agrees with observations of delayed maturation in SSD across a range of functional and structural brain phenotypes. An alternative, not mutually exclusive interpretation is that FC-based BAG is comparatively state-sensitive and may capture compensatory or context-dependent network configurations: more severely affected patients could transiently express connectivity patterns that resemble younger normative profiles, whereas clinical improvement could coincide with reconfiguration toward more typical adult-like organization.

Consistent with the latter possibility, increases in BAG over 12–24 weeks tracked reductions in NSS MoCo and HS NSS which are established markers of aberrant neurodevelopment and can vary with illness course and treatment response [27, 59, 60]. Thus, rising functional BAG over follow-up may reflect a shift toward more normative large-scale connectivity, whether through maturation, partial normalization, or state-dependent stabilization, providing a plausible account for concurrent NSS improvement. In contrast, we did not observe robust associations between BAG and psychopathological symptom severity. This suggests that FC-based BAG may not primarily reflect current symptom burden, but rather broader features of neurodevelopmental deviation, cognitive dysfunction, illness chronicity, or subtle sensorimotor impairment. Previous studies have shown that brain age can differentiate cognitively impaired SSD patients from non-cognitively impaired patients [61], and that brain age is generally elevated in psychotic patients [62]. Additionally, psychopathological symptoms in SSD are heterogeneous and differ in their temporal stability across symptom dimensions [63], which may limit the sensitivity of a global BAG measure to capture symptom-specific variance. However, it is important to keep in mind that this longitudinal result was only seen with the Harvard-Oxford atlas, which either shows an improved signal due to finer granularity of the atlas compared to the MSDL atlas or a more unstable signal due to the smaller longitudinal sample size. Daily antipsychotic dose was not significantly associated with FC-based BAG in the present study. This null finding is notable because it suggests that the observed functional brain-age deviations were not primarily explained by current antipsychotic dose. This is consistent with large-scale structural brain-age work in SSD showing elevated BAG but no association with antipsychotic use or dose [4]. However, this finding should be interpreted cautiously, as antipsychotic exposure has been linked to structural brain changes in longitudinal MRI studies [64] and may also modulate resting-state functional connectivity [65]. Moreover, within this study, daily medication dose does not capture lifetime exposure, medication class, adherence, treatment duration, or medication changes over time. Therefore, the absence of a current-daily dose association does not rule out more complex or cumulative medication-related effects on FC-based BAG. Taken together, these findings support a nuanced view in which structural BAG may more strongly reflect cumulative burden and brain aging patterns, whereas functional BAG may be more sensitive to heterogeneity and short-term clinical state, capturing deviations in network organization that can relate to neurodevelopmental load as well as symptom-linked change [66]. However, more research is needed to confirm these longitudinal results and show their stability.

An important implication of these findings is that the associations of functional BAG with cognition and NSS may converge within a broader neurodevelopmental framework. NSS are widely regarded as markers of aberrant neurodevelopment in SSD and comprise subtle abnormalities in IF, MoCo, and CoMT, among others [67]. Importantly, NSS have been linked to structural brain variation, altered white-matter microstructure, and differences in neural network activity, also observed in healthy adults, suggesting that they reflect dimensional variation in brain organization rather than disease-specific sensorimotor abnormalities alone [68–70]. In SSD, sensorimotor and psychomotor abnormalities have further been associated with psychopathological symptoms, cognition, and global functioning [71]. In the present study, more negative functional BAG was associated with both poorer cognitive performance and greater NSS severity, suggesting that younger-appearing functional brain profiles may capture clinically relevant deviations from normative network maturation. Within this framework, a younger-appearing functional brain should not be interpreted as preserved brain health, but rather may reflect delayed, less differentiated, or atypically organized large-scale functional connectivity. Functional BAG may therefore provide a compact system-level marker of neurodevelopmental heterogeneity that relates to both cognitive dysfunction and NSS in SSD.

Our findings may also be interpreted within the framework of Spatiotemporal Psychopathology (STPP) and the recently proposed imprecision model of schizophrenia [72–75]. Rather than conceptualizing schizophrenia exclusively as accelerated brain aging, these frameworks emphasize disturbances in the temporal and spatial organization of intrinsic brain activity. In particular, the temporal-imprecision model proposes that schizophrenia and psychosis are characterized by reduced precision of neural timing and phase coherence, which may disturb the coordination of neural activity across distributed systems. From this perspective, a younger-appearing functional brain may not only reflect delayed maturation but could also indicate atypical or less differentiated large-scale functional organization that deviates from normative developmental trajectories. Such altered organization could plausibly affect both sensorimotor integration and higher-order cognitive processes, providing a framework for understanding the observed associations between lower functional brain age, higher NSS severity, and poorer cognitive performance. NSS may be consistent with disturbances in cortico-subcortical and sensorimotor network organization, whereas cognitive deficits may reflect related disruptions within association and executive-control networks. Accordingly, FC-based brain-age estimates may provide a systems-level marker of the extent to which intrinsic functional organization deviates from normative maturation, thereby linking neurodevelopmental abnormalities with the altered spatiotemporal organization of brain activity proposed by the STPP framework.

This study has both strengths and limitations: First, it leverages a large, harmonized multi-site lifespan dataset (n ≈ 2200 HCs) to train robust, generalizable rs-fMRI brain-age models. Second, it systematically compares multiple atlases and machine learning algorithms. Third, the study demonstrates strong external validity by applying the models to four independent and SSD cohorts with rigorous harmonization and matching procedures. Finally, it establishes clinical relevance by linking functional brain-age to cognitive deficits, NSS, and symptom trajectories, highlighting its potential as a compact and interpretable biomarker of brain dysmaturation in SSD. Limitations include moderate FC-only MAE compared to multimodal models, relatively short longitudinal follow-up, limited information on cumulative antipsychotic medication exposure, and the absence of direct comparison to structural BAG within the same individuals. Although antipsychotic medication load was examined where available, current dose does not fully capture lifetime exposure, medication class, adherence, or treatment changes over time. Because antipsychotic treatment has been associated with changes in rs-FC [76], medication-related effects cannot be fully disentangled from illness-related, developmental, and state-dependent contributions to FC-based BAG in the present study. Future multimodal normative models that jointly estimate structural and functional BAG will be crucial to disentangle these axes and to test whether FC-BAG improves prediction of long-term outcome or treatment response. By estimating modality-specific BAGs from structural MRI and rs-fMRI, and comparing them with a fused multimodal BAG, future studies could test whether FC-BAG explains clinically relevant variance beyond structural brain aging. Prior work suggests that multimodal models can improve age prediction [77], but that single-modality BAGs may sometimes be more sensitive to specific clinical phenotypes [78]; therefore, future studies should evaluate both model accuracy and clinical utility when predicting long-term outcome or treatment response. An additional consideration is whether rs-FC normative models should be sex-specific. Previous studies have reported sex and sex-by-age effects in rs-FC, suggesting that sex may influence functional-connectivity trajectories across adulthood [79, 80]. In the present study, sex was included as a covariate, but separate sex-specific normative models were not estimated because stratifying the training data by sex would have reduced the effective sample size and may have increased model instability. Future studies with larger balanced samples should directly compare sex-neutral, sex-adjusted, and sex-specific rs-FC normative models.

## Conclusion

These findings suggest that BAG estimates are robust and generalizable across atlases when using simple, well-regularized models, underscoring the feasibility of FC-based brain-age modeling in multi-site clinical settings. Moreover, this study indicated that functional brain age in SSD does not merely reflect accelerated aging but may instead index immaturity in network organization. A shift toward an “older” normative connectivity pattern over time could reflect adaptive plasticity or partial normalization, particularly in cognitive and sensorimotor systems.

## Supplementary information

Supplement

## Data Availability

The inhouse data is not publicly available but can be shared upon reasonable request. The public available datasets can be accessed as follows: CamCAN dataset is available here https://opendata.mrc-cbu.cam.ac.uk/projects/camcan/, AOMIC PIOP1: https://openneuro.org/datasets/ds002785/versions/2.0.0, AOMIC PIOP2: https://openneuro.org/datasets/ds002790/versions/2.0.0, Dallas Lifespan: https://openneuro.org/datasets/ds004856/versions/1.3.0, OASIS-3: https://www.nitrc.org/projects/oasis/, SALD: https://fcon_1000.projects.nitrc.org/indi/retro/sald.html.

## Code availability

Only publicly available software was used for the preprocessing and analysis of this analysis. Fmriprep was used for preprocessing of the MRI data and is available here https://fmriprep.org/en/23.0.1/, connectivity matrices were calculated with nilearn https://nilearn.github.io/stable/index.html. Data was harmonized with https://github.com/rpomponio/neuroHarmonize. Machine learning age regression was calculated with scikit learn https://scikit-learn.org/stable/index.html. Optuna was used for gridsearch https://hub.optuna.org/samplers/grid_search/ statsmodels were used for statistical testing https://www.statsmodels.org/stable/index.html.
