# Supplementary material for: Resting-state brain age is associated with cognitive and sensorimotor abnormalities in schizophrenia spectrum disorders: a validation & longitudinal study
Source: Transl Psychiatry. 2026 Sep 4;16:451. doi: 10.1038/s41398-026-04426-3 (PMC13545253; doi:10.1038/s41398-026-04426-3)
Supplement: Supplementary file 1 [file 41398_2026_4426_MOESM1_ESM.docx]

**Supplement**

# 1 Methods

## 1.1 Data

### 1.1.1 MRI Data Acquisition within the In-House Datasets

**whiteCAT and URBN cohorts:** Structural and functional MRI were acquired at CIMH on a 3 T MAGNETOM Prisma scanner (Siemens Medical Solutions, Erlangen, Germany). T1-weighted structural images were obtained with a 3D MP-RAGE sequence (192 sagittal slices; matrix, 256 × 256; voxel size, 1.0 × 1.0 × 1.0 mm³; TR, 2,300 ms; TE, 3.03 ms; TI, 900 ms; flip angle, 9°). Resting-state fMRI (rsfMRI) used a gradient-echo echo-planar imaging (EPI) sequence (60 slices; matrix, 204 × 204; voxel size, 2.4 × 2.4 × 2.4 mm³; TR, 800 ms; TE, 30 ms; flip angle, 31°; anterior–posterior phase-encoding).

**NSS and BMBF cohorts:** Structural and functional MRI were acquired at CIMH on a 3 T MAGNETOM TIM Trio scanner (Siemens Medical Systems, Erlangen, Germany) equipped with a 32-channel head coil. T1-weighted structural images were obtained with a 3D MP-RAGE sequence (176 sagittal slices; field of view, 256 × 256 mm²; voxel size, 1.0 × 1.0 × 1.0 mm³; TR, 2,530 ms; TE, 3.8 ms; TI, 1,100 ms; flip angle, 7°). Resting-state fMRI used a gradient-echo EPI sequence (60 slices; matrix, 204 × 204; voxel size, 2.4 × 2.4 × 2.4 mm³; TR, 1,790 ms; TE, 28 ms; flip angle, 31°; anterior–posterior phase-encoding).

### 1.1.2 MRI Data Acquisition within the Clinical Publicly Available Datasets

**LA5c-UCLA:** Structural and functional MRI were acquired on one of two 3 T MAGNETOM Trio scanners (Siemens, Erlangen, Germany) at the Ahmanson–Lovelace Brain Mapping Center and the Staglin Center for Cognitive Neuroscience (UCLA). T1-weighted structural images were obtained with a 3D MP-RAGE sequence (176 sagittal slices; field of view, 256 × 256 mm²; voxel size, 1.0 × 1.0 × 1.0 mm³; TR, 1,900 ms; TE, 2.26 ms). Resting-state fMRI used a T2*-weighted echo-planar imaging (EPI) sequence (34 slices; slice thickness, 4 mm; matrix, 64 × 64; field of view, 192 mm; TR, 2,000 ms; TE, 30 ms; flip angle, 90°).

**COBRE:** Data acquisition was coordinated via the Collaborative Informatics and Neuroimaging Suite (COINS) and conducted at the Mind Research Network (Albuquerque, NM) with support from an NIH COBRE grant (5P20RR021938/P20GM103472). Structural MRI was collected on a Siemens 3 T MAGNETOM Trio (Tim) scanner. T1-weighted images were acquired with a multi-echo 3D MP-RAGE sequence (field of view, 256 × 256 mm²; voxel size, 1.0 × 1.0 × 1.0 mm³; TR, 2,530 ms; TEs, 1.64/3.50/5.36/7.22/9.08 ms; TI, 1,200 ms; flip angle, 7°). Resting-state fMRI was acquired axially in interleaved ascending order using single-shot full k-space EPI with ramp-sampling correction aligned to the AC–PC plane (32 slices; matrix, 64 × 64; voxel size, 3 × 3 × 4 mm³; TR, 2,000 ms; TE, 29 ms).

### 1.1.3 MRI Data Acquisition within Healthy Reference Cohort

**CamCAN:** MR data were collected in a 1-h session at MRC-CBSU on a 3T Siemens TIM Trio with a 32-channel head coil. Structural imaging used a 3D T1-weighted MPRAGE (TR/TE/TI = 2250/2.99/900 ms, flip angle 9°, FOV 256×240×192 mm, 1-mm isotropic voxels, GRAPPA=2; 4:32 min). rsfMRI was collected with T2*-weighted GE-EPI while participants rested with eyes closed (261 volumes; 32 axial slices, descending; slice thickness 3.7 mm with 20% gap; TR/TE = 1970/30 ms, flip angle 78°, FOV 192×192 mm, voxel size 3×3×4.44 mm; 8:40 min) ^1^.

**AOMIC-PIOP:** For both PIOP datasets, imaging was performed on the same Philips 3 T system (Philips, Best, the Netherlands) using a 32-channel head coil; the scanner ran the Achieva platform for PIOP1 and was upgraded to Achieva dStream for PIOP2 (earlier digitalization of the MR signal, reducing noise). Resting-state fMRI instructed participants to fixate on a central cross on a gray background (RGB [150,150,150]); eye-tracking was recorded but is not shared. PIOP1 resting state: 6 min (480 volumes, TR = 0.75 s). PIOP2 resting state: 8 min (240 volumes, TR = 2.0 s). Structural T1-weighted scans for both PIOP1 and PIOP2 used 3D MPRAGE with whole-brain coverage, 1 mm isotropic voxels, TR/TE = 8.5/3.9 ms, flip = 8. Functional BOLD EPI parameters: PIOP1 used multiband GE-EPI (MB factor = 3) with whole-brain coverage, 3 mm isotropic voxels, matrix = 80×80, 36 slices, slice gap = 0.3 mm, TR/TE = 750/28 ms, flip = 60°, phase-encode P→A, slice order F→H (sequential), dynamic stabilization “enhanced.” PIOP2 used sequential (non-MB) GE-EPI with whole-brain coverage, 3 mm isotropic voxels, matrix = 80×80, 37 slices, slice gap = 0.3 mm, TR/TE = 2000/28 ms, flip = 76.1°, phase-encode P→A, slice order F→H (sequential), dynamic stabilization “enhanced.” ^2^.

**DLBS:** Participants in epochs 1 and 2 were imaged at the University of Texas Southwestern Medical Center on a 3 T Philips Achieva scanner (Philips Medical Systems, Best, The Netherlands) with an 8-channel head coil. For epoch 3, data were acquired on a newer 3 T Achieva at the same site; to maintain consistency, the original 8-channel coil was used and the acquisition settings were unchanged. Structural images were obtained with a T1-weighted MP-RAGE protocol comprising 160 sagittal slices at 1 mm isotropic resolution (matrix 204 × 256 × 160), TR/TE 8.1/3.7 ms, shot interval 2100 ms, inversion time 1100 ms, and a 12° flip angle. Whole-brain functional data came from a T2*-weighted EPI sequence with 43 interleaved axial slices aligned to the AC–PC plane, SENSE acceleration factor 2, voxel size 3.4 × 3.4 × 3.5 mm (matrix 64 × 64 × 43), field of view 220 × 220 mm, TR/TE 2000/25 ms, and an 80° flip angle. Philips PAR/REC outputs were converted to NIfTI using BIDScoin (Donders Institute, Nijmegen, The Netherlands) ^3^.

**OASIS-3:** All 3T MRI data were acquired at Washington University in St. Louis on Siemens systems—TIM Trio 3 T (two scanners) with 20-channel head coils. Structural imaging comprised high-resolution T1-weighted scans, and functional imaging used T2*-weighted BOLD EPI with whole-brain coverage; representative fMRI settings were TR = 2.5 s, TE = 27 ms, flip angle = 90°, and a matrix size = 64 x 64. Only 3T scanners and the T1w/BOLD acquisitions are described here; other hardware and sequences are omitted by this study design but are available in the original publication ^4^.

**SALD:** All imaging was performed at the Southwest University Center for Brain Imaging on a 3T Siemens Trio (Siemens Medical, Erlangen, Germany). Each participant completed, in a single session, a high-resolution T1-weighted structural scan and a resting-state fMRI scan. The T1-weighted anatomy used an MPRAGE sequence (TR = 1900 ms, TE = 2.52 ms, TI = 900 ms, flip angle = 90°, matrix = 256 × 256, 176 slices, 1.0 mm thickness, 1×1×1 mm³ voxels). Resting-state fMRI consisted of an 8-minute gradient-echo EPI acquisition with 242 volumes while participants lay still with eyes closed (32 slices; TR/TE = 2000/30 ms; flip angle = 90°; field of view = 220 × 220 mm; thickness/gap = 3/1 mm; voxel size = 3.4 × 3.4 × 4 mm³) ^5^.

### 1.1.4 MRI Data Preprocessing

All MRI data were preprocessed using fMRIPrep version 23.2.3 ^6^. Intensity non-uniformity (INU) in the T1-weighted (T1w) images was corrected using N4BiasFieldCorrection^7^, implemented in ANTs version 2.5.0 ^8^, and the resulting T1w image served as the reference throughout the preprocessing workflow. Skull stripping of the T1w reference image was performed using a Nipype implementation of the ANTs antsBrainExtraction.sh workflow, with the OASIS30ANTs template as the target. Segmentation of brain tissue into cerebrospinal fluid (CSF), white matter (WM), and gray matter (GM) compartments was conducted on the skull-stripped T1w images using the FAST algorithm ^9^. For each subject’s single BOLD run, fMRIPrep generated a reference volume for motion correction, estimated head motion with FSL’s MCFLIRT (six rigid‐body parameters and transforms), and co-registered the BOLD reference to the T1w image using FreeSurfer’s boundary-based registration ^10, 11^. From the preprocessed BOLD we derived confounds including framewise displacement and DVARS ^12^, plus global signals from CSF, white matter (WM), and whole brain. Motion estimates and global signals were expanded with temporal derivatives and quadratic terms ^13^. Volumes with FD > 0.5 mm or standardized DVARS > 1.5 were flagged as outliers; edge-band PCA regressors were additionally extracted ^14^. All spatial resamplings were composed into a single step (including distortion and co-registrations) using cubic B-spline interpolation. The quality control was assessed manually in the clinical cohorts by examining the registration of the T1w images and examining the carpet plots of the rsfMRI images. Additionally, all images were dropped that had a higher mean framewise displacement (FD) than 0.5mm.

## 1.2 Estimating Biological Brain Age

All models were trained and evaluated exclusively on the healthy reference cohort to learn a baseline mapping from FC features to chronological age as a proxy for biological age. Each algorithm was wrapped in a scikit-learn Pipeline with StandardScaler for feature scaling. Hyperparameters were optimized per family using optuna exploring the candidate counts shown below; distributions were chosen to be scale-appropriate (log-uniform for multiplicative controls like penalties and learning rates, uniform or discrete sets for others). Nested CV used an outer 5-fold CV repeated 5 times (RepeatedStratifiedKFold, stratified by age decade) and an inner 3-fold CV for tuning. On each outer test split we computed R², MAE, RMSE, and Pearson’s r; 95% CIs were t-based for means (MAE/RMSE/R²) and Fisher z–based for r. For each family we retained the best outer-fold estimator (lowest MAE). After ranking families by mean outer-fold MAE, the top family’s best-fold pipeline was refit on all HC samples to produce the final model. Search spaces (values are inclusive of the sampling support; randint(a,b) samples integers a…b−1; uniform(ℓ,w) samples the continuous interval [ℓ, ℓ+w]): LightGBM (n=15): n_estimators 50–300, learning_rate 1e−3–1e−1 (log-uniform), num_leaves 10–200, max_depth {2,3,4}, min_child_samples 10–80, subsample 0.7–0.9, colsample_bytree 0.6–1.0, reg_lambda 1e−3–3.0 (log-uniform), reg_alpha 1e−2–1.0 (log-uniform), metric {l1}. XGBoost (n=15): n_estimators 50–300, learning_rate 1e−3–1e−1 (log-uniform), max_depth {2,3,4}, min_child_weight 0.3–8.0 (log-uniform), subsample 0.7–0.9, colsample_bytree 0.6–1.0, reg_lambda 1e−3–5.0 (log-uniform), reg_alpha 1e−4–1.0 (log-uniform), gamma 1e−3–1.0 (log-uniform). RandomForest (n=15): n_estimators 50–300, max_depth {2,3,4}, min_samples_leaf 1–5, min_samples_split 2–10, max_features {sqrt }, criterion {squared_error }, max_samples 0.7–0.9. SVR (n=15): RBF kernel, C 1e−1–1e2 (log-uniform), epsilon 1e−2–5e−1 (log-uniform), gamma {scale}. ElasticNet (n=15): alpha 1e−4–1.0 (log-uniform), l1_ratio 0.0–1.0 (uniform). Ridge (n=15): alpha 1e−4–10.0 (log-uniform). Lasso (n=15): alpha 1e−4–1.0 (log-uniform).

# 2 Results

**Table 1.** Descriptive statistics of the healthy reference cohort.

| Site | n | Age | Mean FD | Female Count |
| --- | --- | --- | --- | --- |
| CamCan | 588 | 52.94 (SD 18.5) | 0.24 (SD .1) | 290 |
| AOMIC 1 | 203 | 22.21 (SD 1.8) | 0.11 (SD .04) | 117 |
| AOMIC 2 | 214 | 21.93 (SD 1.8) | 0.13 (SD .04) | 124 |
| DLBS | 296 | 60.46 (SD 17.73) | 0.2 (SD .09) | 187 |
| OASIS | 489 | 64.96 (SD 9.18) | 0.25 (SD .1) | 305 |
| SALD | 432 | 42.93 (SD 16.93) | 0.18 (SD .09) | 267 |

**Table 2.** Descriptive statistics of the clinical cohorts that were used for the group difference analysis. * Indicate a statistical significant difference between HC and SSD subjects. Age and mean FD were calculated using a *t*-test and sex was calculated using Chi-squared test.

| Site | diagnosis | n | Age | Mean FD | Female Count |
| --- | --- | --- | --- | --- | --- |
| UCLA | HC | 45 | 35.58 (SD 9.67) | 0.16 (SD .08)* | 9 |
|  | SSD | 45 | 36.33 (SD 8.92) | 0.22 (SD .09)* | 10 |
| COBRE | HC | 58 | 36.79 (SD 11.83) | 0.25 (SD .11) | 10 |
|  | SSD | 59 | 36.42 (SD 13.25) | 0.29 (SD .11) | 10 |
| whiteCAT/URBN | HC | 33 | 34.04 (SD 10.32) | 0.15 (SD .09) | 14 |
|  | SSD | 83 | 35.43 (SD 11.85) | 0.16 (SD .08) | 26 |
| whiteCAT follow-up | HC | 0 | - | - | - |
|  | SSD | 57 | 36.69 (SD 12.64) | 0.15 (SD 0.07) | 19 |
| NSS/BMBF | HC | 103 | 33.11 (SD 10.28)* | 0.18 (SD .07)* | 50 |
|  | SSD | 103 | 37.69 (SD 11.13)* | 0.24 (SD .11)* | 51 |
| NSS follow-up | HC | 0 | - | - | - |
|  | SSD | 31 | 37.13 (SD 0.1) | 0.25 (0.1) | 11 |

**Table 3.** Descriptive and clinical information about the clinical cohorts that were used to test the clinical associations with the BAG. This sample only includes SSD patients from the studies whiteCAT and NSS.

| Clinical Variable | whiteCAT (n=90) | NSS(n=103) |
| --- | --- | --- |
| TMT | 109.44 (SD 91.8) | 104.36 (SD 59.59) |
| CF | 19.84 (SD 6.43) | 18.49 (SD 13.6) |
| DSST | 56.39 (SD 18.86) | 51.27 (SD 27.29) |
| Duration Illness (years) | 10.66 (SD 10.88) | 9.02 (SD 9.86) |
| NSS HS | 3.21 (SD 2.22) | 2.88 (SD 1.71) |
| NSS IF | 4.4 (SD 1.89) | 2.45 (SD 1.42) |
| NSS CoMT | 3.43 (SD 2.09) | 3.32 (SD 2.34) |
| NSS MoCo | 7.38 (SD 4.49) | 7.33 (SD 3.67) |
| NSS RLSpO | 3.33 (SD 2.71) | 2.41 (SD 2.25) |
| NSS Total | 19.79 (SD 9.23) | 18.39 (SD 7.89) |
| Olanzapine Equivalent | 12.94 (SD 9.45) | 18.94 (SD 18.48) |
| PANSS General | 32.28 (SD 7.87) | 35.56 (SD 11.49) |
| PANSS Negative | 17.37 (SD 6.85) | 16.53 (SD 7.52) |
| PANSS Positive | 15.3 (SD 5.25) | 15.73 (SD 7.23) |
| PANSS Total | 64.94 (SD 16.21) | 67.73 (SD 22.13) |

**Table 4.** Clinical characteristics of baseline and follow-up examinations of the two clinical in-house cohorts. Follow-up was six months after the first visit in the NSS cohort, and three months after the first visit in the whiteCAT cohort.

| Clinical Variable | whiteCAT (n=57) | NSS (n=31) |
| --- | --- | --- |
| TMT baseline | 91.8 (SD 48.55) | 97.46 (SD 44.38) |
| Follow-up | 89.07 (SD 53.17) | 83.63 (SD 34.58) |
| CF baseline | 20.84 (SD 5.93) | 18.49 (SD 6.31) |
| Follow-up | 19.44 (SD 5.27) | 22.47 (SD 11.78) |
| DSST baseline | 58.41 (SD 18.32) | 53.73 (SD 15.62) |
| Follow-up | 64.8 (SD 18.77) | 49.07 (SD 42.98) |
| NSS HS baseline | 3.14 (SD 2.19) | 3.13 (SD 1.73) |
| Follow-up | 2.64 (SD 1.9) | 2.43 (SD 1.79) |
| NSS IF baseline | 4.28 (SD 1.4) | 2.463 (SD 1.6) |
| Follow-up | 4.23 (SD 1.74) | 2.23 (SD 1.22) |
| NSS CoMT baseline | 3.23 (SD 1.89) | 3.33 (SD 2.06) |
| Follow-up | 2.75 (SD 2.59) | 2.1 (SD 1.81) |
| NSS MoCo baseline | 6.7 (SD 3.73) | 7.37 (SD 3.37) |
| Follow-up | 6.12 (SD 4.23) | 6.07 (SD 3.65) |
| NSS RLSpO baseline | 3.03 (SD 2.35) | 2.3 (SD 1.7) |
| Follow-up | 2.73 (SD 2.29) | 1.5 (SD 1.41) |
| NSS Total baseline | 18.72 (SD 7.25) | 18.76 (SD 6.04) |
| Follow-up | 16.81 (SD 9.27) | 13.9 (SD 6.89) |
| PANSS General baseline | 31.23 (SD 7.76) | 38.37 (SD 13.88) |
| Follow-up | 28.11 (SD 7.55) | 30.27 (SD 9.01) |
| PANSS Negative baseline | 16.63 (SD 6.79) | 18.27 (SD 8.06) |
| Follow-up | 15.09 (SD 6.85) | 14.4 (SD 4.96) |
| PANSS Positive baseline | 14.46 (SD 4.89) | 15.87 (SD 8.19) |
| Follow-up | 12.23 (SD 4.38) | 10.83 (SD 5.15) |
| PANSS Total baseline | 62.31 (SD 15.71) | 72.5 (SD 25.3) |
| Follow-up | 55.42 (SD 14.46) | 54.8 (SD 16.39) |

**Table 5.** Results of the group comparisons of BAG between HC and SSD participants for the MSDL atlas. The tables report Cohen’s *d*, Hedges’ *g*, and Welch’s *t*-test results separately for each cohort. q values refer to FDR-corrected p values.

| Site | n HC | Mean HC | SD HC | n SSD | Mean SSD | SD SSD | Mean diff (SSD − HC) | Cohen's d | Hedges' g | t (Welch) | q |
| --- | --- | --- | --- | --- | --- | --- | --- | --- | --- | --- | --- |
| UCLA | 45 | 6.9e-17 | 1 | 45 | 0.59 | 1.3 | 0.6 | 0.52 | 0.51 | 2.446 | 0.02 |
| COBRE | 58 | 1.34e-17 | 1 | 59 | 0.12 | 1.14 | 0.12 | 0.11 | 0.11 | 0.5857 | 0.56 |
| whiteCAT  URBAN | 33 | 1.01e-17 | 1 | 83 | 0.64 | 1.36 | 0.64 | 0.50 | 0.50 | 2.791 | 0.01 |
| NSS  BMBF | 103 | 4.74e-17 | 1 | 103 | 0.63 | 1.54 | 0.63 | 0.48 | 0.48 | 3.467 | 0.002 |

**Table 6.** Results of the group comparisons of BAG between HC and SSD participants for the Harvard-Oxford atlas. The tables report Cohen’s d, Hedges’ g, and Welch’s t-test results separately for each cohort. q values refer to FDR-corrected p values.

| Site | n HC | Mean HC | SD HC | n SSD | Mean SSD | SD SSD | Mean diff (SSD − HC) | Cohen's d | Hedges' g | t (Welch) | q |
| --- | --- | --- | --- | --- | --- | --- | --- | --- | --- | --- | --- |
| UCLA | 45 | 3.7e-18 | 1 | 45 | 0.4 | 0.94 | 0.4 | 0.41 | 0.41 | 1.94 | 0.05 |
| COBRE | 59 | -7.15e-17 | 1 | 59 | 0.74 | 1.18 | 0.74 | 0.67 | 0.67 | 3.66 | 5.5e5 |
| whiteCAT  URBAN | 33 | 5.05e-17 | 1 | 83 | 0.94 | 1.42 | 0.94 | 0.72 | 0.71 | 4.04 | 2.4e4 |
| NSS  BMBF | 102 | 1.08e-16 | 1 | 103 | 0.9 | 1.47 | 0.93 | 0.72 | 0.72 | 5.15 | 2.7e7 |

**Table 7.** Results of the post-hoc Welch’s t-tests in the whiteCAT and NSS cohorts, comparing individuals with MSDL-based BAG values at least 0.5 SD below the mean (“young”) or at least 0.5 SD above the mean (“old”). q refers to FDR corrected p-values.

| Site | Variable | N Young | N Old | Young mean | Old mean | Diff | t | q |
| --- | --- | --- | --- | --- | --- | --- | --- | --- |
| whiteCAT/NSS | TMT | 54 | 85 | 114.4 | 85.17 | -29.28 | -3.038 | 0.01814 |
|  | CF | 54 | 86 | 19.41 | 19.4 | -0.01206 | -0.006772 | 0.9946 |
|  | DSST | 54 | 85 | 53.15 | 58.02 | 4.875 | 1.384 | 0.5004 |
|  | DOI | 54 | 86 | 11.44 | 6.326 | -5.119 | -3.12 | 0.01814 |
|  | NSS HS | 54 | 86 | 3.185 | 2.988 | -0.1968 | -0.6098 | 0.6939 |
|  | NSS IF | 54 | 86 | 3.315 | 3.07 | -0.245 | -0.844 | 0.6939 |
|  | NSS CoMT | 54 | 86 | 3.556 | 3.07 | -0.4858 | -1.289 | 0.5004 |
|  | NSS MoCo | 54 | 86 | 8 | 6.093 | -1.907 | -2.979 | 0.01814 |
|  | NSS RLSpO | 54 | 86 | 2.889 | 2.581 | -0.3075 | -0.836 | 0.6939 |
|  | NSS Total | 54 | 86 | 20.15 | 16.95 | -3.195 | -2.57 | 0.04342 |
|  | OLZ | 54 | 86 | 16.36 | 17.86 | 1.498 | 0.5916 | 0.6939 |
|  | PANSS G | 54 | 86 | 34.26 | 33.12 | -1.143 | -0.6768 | 0.6939 |
|  | PANSS N | 54 | 86 | 16.87 | 17.05 | 0.1761 | 0.1368 | 0.9551 |
|  | PANSS P | 54 | 86 | 15.37 | 14.73 | -0.6378 | -0.6003 | 0.6939 |
|  | PANSS Total | 54 | 86 | 66.37 | 64.86 | -1.51 | -0.4468 | 0.7567 |
| whiteCAT | TMT | 25 | 39 | 102.3 | 88.85 | -13.47 | -0.9919 | 0.7358 |
|  | CF | 25 | 40 | 19.48 | 20.85 | 1.37 | 0.9552 | 0.7358 |
|  | DSST | 25 | 39 | 54.8 | 60.44 | 5.636 | 1.191 | 0.7207 |
|  | DOI | 25 | 40 | 14.32 | 5.8 | -8.52 | -3.448 | 0.02293 |
|  | NSS HS | 25 | 40 | 3.08 | 3.2 | 0.12 | 0.2212 | 0.8848 |
|  | NSS IF | 25 | 40 | 4.32 | 3.825 | -0.495 | -1.298 | 0.7207 |
|  | NSS CoMT | 25 | 40 | 3.28 | 3.1 | -0.18 | -0.3936 | 0.8699 |
|  | NSS MoCo | 25 | 40 | 8.16 | 5.95 | -2.21 | -2.27 | 0.2067 |
|  | NSS RLSpO | 25 | 40 | 3.24 | 3.05 | -0.19 | -0.3083 | 0.8756 |
|  | NSS Total | 25 | 40 | 20.36 | 17.3 | -3.06 | -1.737 | 0.4394 |
|  | OLZ | 25 | 40 | 14.74 | 13.68 | -1.067 | -0.4401 | 0.8699 |
|  | PANSS G | 25 | 40 | 31.04 | 32.38 | 1.335 | 0.7629 | 0.7932 |
|  | PANSS N | 25 | 40 | 16.76 | 16.95 | 0.19 | 0.1085 | 0.914 |
|  | PANSS P | 25 | 40 | 13.96 | 14.8 | 0.84 | 0.7188 | 0.7932 |
|  | PANSS Total | 25 | 40 | 61.76 | 64.12 | 2.365 | 0.6186 | 0.8083 |
| NSS | TMT | 29 | 46 | 124.9 | 82.04 | -42.85 | -3.18 | 0.03927 |
|  | CF | 29 | 46 | 19.34 | 18.13 | -1.214 | -0.3923 | 0.8032 |
|  | DSST | 29 | 46 | 51.72 | 55.98 | 4.254 | 0.8266 | 0.514 |
|  | DOI | 29 | 46 | 8.966 | 6.783 | -2.183 | -1.032 | 0.5104 |
|  | NSS HS | 29 | 46 | 3.276 | 2.804 | -0.4715 | -1.234 | 0.5104 |
|  | NSS IF | 29 | 46 | 2.448 | 2.413 | -0.03523 | -0.106 | 0.9307 |
|  | NSS CoMT | 29 | 46 | 3.793 | 3.043 | -0.7496 | -1.283 | 0.5104 |
|  | NSS MoCo | 29 | 46 | 7.862 | 6.217 | -1.645 | -1.915 | 0.3361 |
|  | NSS RLSpO | 29 | 46 | 2.586 | 2.174 | -0.4123 | -0.9763 | 0.5104 |
|  | NSS Total | 29 | 46 | 19.97 | 16.65 | -3.313 | -1.872 | 0.3361 |
|  | OLZ | 29 | 46 | 17.76 | 21.5 | 3.741 | 0.8949 | 0.5104 |
|  | PANSS G | 29 | 46 | 37.03 | 33.76 | -3.274 | -1.223 | 0.5104 |
|  | PANSS N | 29 | 46 | 16.97 | 17.13 | 0.1649 | 0.08737 | 0.9307 |
|  | PANSS P | 29 | 46 | 16.59 | 14.67 | -1.912 | -1.132 | 0.5104 |
|  | PANSS Total | 29 | 46 | 70.34 | 65.5 | -4.845 | -0.9131 | 0.5104 |

**Table 8.** Results of the post-hoc Welch’s t-tests in the whiteCAT and NSS cohorts, comparing individuals with Harvard-Oxford-based BAG values at least 0.5 SD below the mean (“young”) or at least 0.5 SD above the mean (“old”). q refers to FDR corrected p-values.

| Site | Variable | N Young | N Old | Young mean | Old mean | Diff | t | q |
| --- | --- | --- | --- | --- | --- | --- | --- | --- |
| whiteCAT/NSS | TMT | 43 | 91 | 138.3 | 88.83 | -49.45 | -2.596 | 0.0466 |
|  | CF | 44 | 91 | 19.07 | 18.77 | -0.299 | -0.162 | 0.9061 |
|  | DSST | 43 | 91 | 49.23 | 53.89 | 4.658 | 1.104 | 0.4923 |
|  | DOI | 44 | 91 | 15.27 | 6.571 | -8.701 | -3.971 | 0.002957 |
|  | NSS HS | 45 | 91 | 3.778 | 2.692 | -1.085 | -3.002 | 0.02723 |
|  | NSS IF | 45 | 91 | 4.089 | 3.011 | -1.078 | -2.821 | 0.03082 |
|  | NSS CoMT | 45 | 91 | 3.844 | 3.011 | -0.8335 | -1.826 | 0.1546 |
|  | NSS MoCo | 45 | 91 | 8.244 | 6.604 | -1.64 | -2.107 | 0.09569 |
|  | NSS RLSpO | 45 | 91 | 2.822 | 2.758 | -0.06398 | -0.1184 | 0.9061 |
|  | NSS Total | 45 | 91 | 21.58 | 17.46 | -4.116 | -2.346 | 0.06545 |
|  | OLZ | 45 | 91 | 18.77 | 14.53 | -4.243 | -1.057 | 0.4923 |
|  | PANSS G | 45 | 91 | 33.73 | 34.16 | 0.4315 | 0.2247 | 0.9061 |
|  | PANSS N | 45 | 91 | 16.87 | 17.52 | 0.6498 | 0.5074 | 0.9061 |
|  | PANSS P | 45 | 91 | 15.07 | 15.37 | 0.307 | 0.2708 | 0.9061 |
|  | PANSS Total | 45 | 91 | 65.51 | 67.02 | 1.511 | 0.4258 | 0.9061 |
| whiteCAT | TMT | 20 | 40 | 153.5 | 93.19 | -60.31 | -1.601 | 0.3109 |
|  | CF | 21 | 40 | 20.24 | 19.43 | -0.8131 | -0.3583 | 0.9433 |
|  | DSST | 20 | 40 | 48.35 | 57.15 | 8.8 | 1.612 | 0.3109 |
|  | DOI | 21 | 40 | 16.38 | 6.8 | -9.581 | -2.97 | 0.08799 |
|  | NSS HS | 22 | 40 | 4 | 2.675 | -1.325 | -2.269 | 0.1552 |
|  | NSS IF | 22 | 40 | 5.318 | 4.15 | -1.168 | -2.238 | 0.1552 |
|  | NSS CoMT | 22 | 40 | 4.273 | 3.025 | -1.248 | -1.94 | 0.2298 |
|  | NSS MoCo | 22 | 40 | 7.682 | 7.625 | -0.05682 | -0.04284 | 0.9722 |
|  | NSS RLSpO | 22 | 40 | 3.227 | 3.5 | 0.2727 | 0.3398 | 0.9433 |
|  | NSS Total | 22 | 40 | 22.05 | 19.57 | -2.47 | -0.9126 | 0.6631 |
|  | OLZ | 22 | 40 | 12.99 | 10.78 | -2.217 | -0.8545 | 0.6631 |
|  | PANSS G | 22 | 40 | 32.5 | 32.58 | 0.075 | 0.03507 | 0.9722 |
|  | PANSS N | 22 | 40 | 18 | 18.43 | 0.425 | 0.232 | 0.9433 |
|  | PANSS P | 22 | 40 | 16.55 | 14.95 | -1.595 | -1.053 | 0.6412 |
|  | PANSS Total | 22 | 40 | 67.05 | 65.95 | -1.095 | -0.2487 | 0.9433 |
| NSS | TMT | 23 | 51 | 125 | 85.41 | -39.63 | -2.725 | 0.07527 |
|  | CF | 23 | 51 | 18 | 18.25 | 0.2549 | 0.09376 | 0.9256 |
|  | DSST | 23 | 51 | 50 | 51.33 | 1.333 | 0.2144 | 0.9256 |
|  | DOI | 23 | 51 | 14.26 | 6.392 | -7.869 | -2.586 | 0.07546 |
|  | NSS HS | 23 | 51 | 3.565 | 2.706 | -0.8593 | -1.907 | 0.1618 |
|  | NSS IF | 23 | 51 | 2.913 | 2.118 | -0.7954 | -2.006 | 0.1606 |
|  | NSS CoMT | 23 | 51 | 3.435 | 3 | -0.4348 | -0.6748 | 0.7562 |
|  | NSS MoCo | 23 | 51 | 8.783 | 5.804 | -2.979 | -3.417 | 0.02369 |
|  | NSS RLSpO | 23 | 51 | 2.435 | 2.176 | -0.2583 | -0.3518 | 0.9095 |
|  | NSS Total | 23 | 51 | 21.13 | 15.8 | -5.327 | -2.285 | 0.1122 |
|  | OLZ | 23 | 51 | 24.3 | 17.47 | -6.827 | -0.934 | 0.6743 |
|  | PANSS G | 23 | 51 | 34.91 | 35.41 | 0.4987 | 0.1601 | 0.9256 |
|  | PANSS N | 23 | 51 | 15.78 | 16.8 | 1.021 | 0.5675 | 0.7818 |
|  | PANSS P | 23 | 51 | 13.65 | 15.71 | 2.054 | 1.276 | 0.4436 |
|  | PANSS Total | 23 | 51 | 64.04 | 67.86 | 3.819 | 0.6953 | 0.7562 |

References

1.	Taylor JR, Williams N, Cusack R, Auer T, Shafto MA, Dixon M *et al.* The Cambridge Centre for Ageing and Neuroscience (Cam-CAN) data repository: Structural and functional MRI, MEG, and cognitive data from a cross-sectional adult lifespan sample. *neuroimage* 2017; **144:** 262-269.

2.	Snoek L, van der Miesen MM, Beemsterboer T, Van Der Leij A, Eigenhuis A, Steven Scholte H. The Amsterdam Open MRI Collection, a set of multimodal MRI datasets for individual difference analyses. *Scientific data* 2021; **8**(1)**:** 85.

3.	Park DC, Hennessee JP, Smith ET, Chan MY, Chen X, Dakanali M *et al.* The dallas lifespan brain study: A comprehensive adult lifespan data set of brain and cognitive aging. *Scientific Data* 2025; **12**(1)**:** 846.

4.	LaMontagne PJ, Benzinger TL, Morris JC, Keefe S, Hornbeck R, Xiong C *et al.* OASIS-3: longitudinal neuroimaging, clinical, and cognitive dataset for normal aging and Alzheimer disease. *medrxiv* 2019**:** 2019.2012. 2013.19014902.

5.	Wei D, Zhuang K, Chen Q, Yang W, Liu W, Wang K *et al.* Structural and functional MRI from a cross-sectional Southwest University Adult life span Dataset (SALD). bioRxiv: 177279. 2018.

6.	Esteban O, Markiewicz CJ, Blair RW, Moodie CA, Isik AI, Erramuzpe A *et al.* fMRIPrep: a robust preprocessing pipeline for functional MRI. *Nat Methods* 2019; **16**(1)**:** 111-116.

7.	Tustison NJ, Avants BB, Cook PA, Zheng Y, Egan A, Yushkevich PA *et al.* N4ITK: improved N3 bias correction. *IEEE Trans Med Imaging* 2010; **29**(6)**:** 1310-1320.

8.	Avants BB, Epstein CL, Grossman M, Gee JC. Symmetric diffeomorphic image registration with cross-correlation: evaluating automated labeling of elderly and neurodegenerative brain. *Med Image Anal* 2008; **12**(1)**:** 26-41.

9.	Zhang Y, Brady M, Smith S. Segmentation of brain MR images through a hidden Markov random field model and the expectation-maximization algorithm. *IEEE Trans Med Imaging* 2001; **20**(1)**:** 45-57.

10.	Jenkinson M, Bannister P, Brady M, Smith S. Improved optimization for the robust and accurate linear registration and motion correction of brain images. *Neuroimage* 2002; **17**(2)**:** 825-841.

11.	Greve DN, Fischl B. Accurate and robust brain image alignment using boundary-based registration. *Neuroimage* 2009; **48**(1)**:** 63-72.

12.	Power JD, Mitra A, Laumann TO, Snyder AZ, Schlaggar BL, Petersen SE. Methods to detect, characterize, and remove motion artifact in resting state fMRI. *neuroimage* 2014; **84:** 320-341.

13.	Satterthwaite TD, Elliott MA, Gerraty RT, Ruparel K, Loughead J, Calkins ME *et al.* An improved framework for confound regression and filtering for control of motion artifact in the preprocessing of resting-state functional connectivity data. *Neuroimage* 2013; **64:** 240-256.

14.	Patriat R, Reynolds RC, Birn RM. An improved model of motion-related signal changes in fMRI. *NeuroImage* 2017; **144:** 74-82.
